# A Novel Anti-Inflammatory and Pro-Resolving Role for Resolvin D1 in Acute Cigarette Smoke-Induced Lung Inflammation

**DOI:** 10.1371/journal.pone.0058258

**Published:** 2013-03-06

**Authors:** Hsi-Min Hsiao, Ramil E. Sapinoro, Thomas H. Thatcher, Amanda Croasdell, Elizabeth P. Levy, Robert A. Fulton, Keith C. Olsen, Stephen J. Pollock, Charles N. Serhan, Richard P. Phipps, Patricia J. Sime

**Affiliations:** 1 Department of Pathology and Laboratory Medicine, University of Rochester, Rochester, New York, United States of America; 2 Lung Biology and Disease Program, University of Rochester, Rochester, New York, United States of America; 3 Division of Pulmonary and Critical Care Medicine, University of Rochester, Rochester, New York, United States of America; 4 Department of Environmental Medicine, University of Rochester, Rochester, New York, United States of America; 5 Department of Microbiology and Immunology, University of Rochester, Rochester, New York, United States of America; 6 Center for Experimental Therapeutics and Reperfusion Injury, Department of Anesthesiology, Perioperative, and Pain Medicine, Brigham and Women’s Hospital, Harvard Institutes of Medicine and Harvard Medical School, Boston, Massachusetts, United States of America; Institute of Lung Biology and Disease (iLBD), Helmholtz Zentrum München, Germany

## Abstract

**Introduction:**

Cigarette smoke is a profound pro-inflammatory stimulus that contributes to acute lung injuries and to chronic lung disease including COPD (emphysema and chronic bronchitis). Until recently, it was assumed that resolution of inflammation was a passive process that occurred once the inflammatory stimulus was removed. It is now recognized that resolution of inflammation is a bioactive process, mediated by specialized lipid mediators, and that normal homeostasis is maintained by a balance between pro-inflammatory and pro-resolving pathways. These novel small lipid mediators, including the resolvins, protectins and maresins, are bioactive products mainly derived from dietary omega-3 and omega-6 polyunsaturated fatty acids (PUFA). We hypothesize that resolvin D1 (RvD1) has potent anti-inflammatory and pro-resolving effects in a model of cigarette smoke-induced lung inflammation.

**Methods:**

Primary human lung fibroblasts, small airway epithelial cells and blood monocytes were treated with IL-1β or cigarette smoke extract in combination with RvD1 *in vitro*, production of pro-inflammatory mediators was measured. Mice were exposed to dilute mainstream cigarette smoke and treated with RvD1 either concurrently with smoke or after smoking cessation. The effects on lung inflammation and lung macrophage populations were assessed.

**Results:**

RvD1 suppressed production of pro-inflammatory mediators by primary human cells in a dose-dependent manner. Treatment of mice with RvD1 concurrently with cigarette smoke exposure significantly reduced neutrophilic lung inflammation and production of pro-inflammatory cytokines, while upregulating the anti-inflammatory cytokine IL-10. RvD1 promoted differentiation of alternatively activated (M2) macrophages and neutrophil efferocytosis. RvD1 also accelerated the resolution of lung inflammation when given after the final smoke exposure.

**Conclusions:**

RvD1 has potent anti-inflammatory and pro-resolving effects in cells and mice exposed to cigarette smoke. Resolvins have strong potential as a novel therapeutic approach to resolve lung injury caused by smoke and pulmonary toxicants.

## Introduction

Cigarette smoke is a potent pro-inflammatory stimulus and a major risk factor for many diseases having an inflammatory component including cardiovascular disease, stroke and lung cancer. Tobacco smoking is the primary risk factor for chronic obstructive pulmonary disease (COPD) emphysema and chronic bronchitis, and is the fifth leading cause of death worldwide [1]. Inflammation is a key factor contributing to lung diseases caused by smoking. Until recently, it was assumed that resolution of inflammation was a passive process that occurred once the inflammatory stimulus was removed. It is now recognized that resolution of inflammation is a bioactive process, mediated by specialized lipid mediators, and that normal homeostasis is maintained by a balance between pro-inflammatory and pro-resolving pathways [2].

Several classes of pro-resolving mediators have been identified, including resolvins, protectins, and maresins [3]. These specialized lipid mediators are derived via enzymatic processing from dietary omega-3 polyunsaturated fatty acids (ω-3-PUFAs), including eicosapentaenoic acid (EPA) and docosahexaenoic acid (DHA). Several of these mediators have anti-inflammatory and pro-resolving activity in pre-clinical disease models such as peritonitis [4], colitis [5], dermal inflammation [6] and asthma [7]. Protectin D1 and resolvin E1 reduce airway hyperresponsiveness and inflammation in animal models of pneumonia and asthma [8],[9]. Furthermore, diets rich in ω-3-PUFAs appear to have beneficial effects in lung diseases such as COPD and asthma [10], [11]. These findings support the idea that pro-resolving lipid mediators in the lung could play a key role in controlling inflammation and return to homeostasis.

Many cell types in the lung respond to cigarette smoke by producing pro-inflammatory mediators. We have previously reported that lung fibroblasts play a significant role in orchestrating inflammatory responses, and respond to cigarette smoke by increasing expression of COX-2 and production of pro-inflammatory prostaglandins and other pro-inflammatory mediators [12]. Macrophages also respond to cigarette smoke by producing pro-inflammatory mediators [13]. These pro-inflammatory mediators recruit immune cells such as neutrophils and additional macrophages and stimulate nearby epithelial and endothelial cells resulting in inflammation and tissue damage.

Interestingly, lung macrophages play a dual role in the response to cigarette smoke. Classically activated, so-called M1 macrophages, express inducible nitric oxide synthase (iNOS) and tumor necrosis factor-α (TNF-α), and are generally thought to be pro-inflammatory. Recent research has demonstrated the importance of alternatively activated, or M2 macrophages, which are activated by Th2 cytokines such as IL-4 and IL-13, or the immunosuppressive cytokines IL-10 and TGF-β [14]. M2 lung macrophages play an essential role in the clearance of inhaled particles, pathogens and apoptotic cells including neutrophils (efferocytosis) [15]–[17]. Studies have shown that cigarette smoking is associated with defects in lung macrophage function [18] and macrophages isolated from COPD patients had a reduced ability to clear apoptotic neutrophils [19], [20], suggesting that macrophage defects are a key pathogenic feature of the disease. Thus, fibroblasts and macrophages are not only useful as *in vitro* models to evaluate new anti-inflammatory and pro-resolving molecules, but are highly relevant to *in vivo* therapeutic outcomes.

Resolvin D1 (RvD1, 7S,8R,17S-trihydroxy-4Z,9E,11E,13Z,15E,19Z-docosahexaenoic acid) is a derivative of DHA [4] with potent anti-inflammatory and pro-resolving properties. The 17R epimer of RvD1 (17R-RvD1) is produced via an alternative biochemical pathway but has similar *in vitro* and *in vivo* activity and uses the same receptors as RvD1. Notably, 17R-RvD1 is resistant to inactivation by the endogenous enzyme 15-prostaglandin dehydrogenase/eicosanoid oxidoreductase (15-PDGH) and potentially has a longer duration of effect *in vivo* [21]. RvD1 and 17R-RvD1 inhibit neutrophilic inflammation and promote efferocytosis in several disease models [22]–[25]. RvD1 also promotes M2 differentiation in mouse models of peritonitis and obesity-induced adipose tissue inflammation and insulin resistance [26], [27].

Here, we investigated the impact of RvD1 on the production of pro-inflammatory mediators by human primary lung fibroblasts, epithelial cells and blood-derived macrophages stimulated with cigarette smoke extract or IL-1β and in a mouse model of acute cigarette smoke (CS)-induced lung inflammation. RvD1 has potent anti-inflammatory effects *in vitro* and *in vivo*, and inhibition of inflammation is associated with increased differentiation and function of M2 lung macrophages. These results suggest that RvD1 may be a potential new therapeutic for cigarette smoke-induced lung injury and other inflammatory lung diseases.

## Materials and Methods

### Ethics Statement

All animal procedures were approved and supervised by the University of Rochester University Committee on Animal Resources (UCAR permit number 2007-127). For euthanasia, mice were anesthetized with an intraperitoneal injection of avertin (2,2,2-tribromoethanol, 250 mg/kg), followed by exsanguination and removal of the lungs for analysis, and all efforts were made to minimize suffering. Primary human lung fibroblasts were derived from culture of tissue explants following biopsy specimens from patients undergoing lung resection surgery for hamartoma, a benign lung tumor. The fibroblasts were grown tissue from anatomically normal areas of the specimen distal to the lesion. Human blood monocytes were isolated from peripheral blood collected from healthy donors. All blood and tissue donors provided informed written consent as described in study protocols approved by the University of Rochester Institutional Review Board and conforming to the Helsinki Declarations. Primary human small airway epithelial cell cultures were obtained from a commercial source.

### Cell Culture and Analysis of Pro-inflammatory Mediators

Primary human lung fibroblast cultures were established from lung tissue explants as previously described [28] and used at early passage (P3–P9). Primary human small airway epithelial cells (SAEC) were purchased from a commercial vendor (Lonza, Allendale, NJ) and cultured in small airway epithelial cell growth medium supplemented as recommended by the supplier. Blood monocytes were cultured in RPMI 1640 as described [29].

Serum starvation was performed to reduce the effect of serum components on induction of cyclooxygenase-2 (COX-2) and other inflammatory mediators. Aqueous cigarette smoke extract (CSE) was prepared as previously described [30]. Briefly, smoke from two reference cigarettes (1R3F, University of Kentucky) was bubbled through 20 ml of serum free medium. This is arbitrarily defined as “100% CSE.” To adjust for batch-to-batch variation, the average absorbance at 320 nm for several batches of CSE was measured and determined to be 0.65 absorbance units. Thereafter, CSE preparations are diluted as needed so that 100% CSE has a standard OD_320_ = 0.65. The pH of the CSE is adjusted to 7.4, and the CSE is then sterile filtered with a 0.45-µm filter (25-mm Acrodisc; Pall, Ann Arbor, MI). CSE was freshly prepared for each experiment and diluted with serum-free MEM immediately before use. Cultures were pre-treated with 10 nM, 100 nM or 1 µM RvD1 or vehicle control solution (0.1% ethanol) for 24 hours prior to the addition of 1% or 5%CSE or 1 ng/ml IL-1β (R&D Systems, Minneapolis, MN) for an additional 24 hours. As the RvD1 was supplied in ethanol, vehicle control cultures were treated with the same final concentration of ethanol (0.1%). Inflammatory mediators were determined in cell culture supernatants as described below. Cell lysates were collected and analyzed by western blotting using an antibody specific for COX-2 (Cayman Chemical, Ann Arbor, MI) and or GAPDH (Abcam, Cambridge, MA), which serves as a normalization control.

### Cigarette Smoke Exposures

Adult female C57BL/6J mice were purchased from The Jackson Laboratory (Bar Harbor, ME) and used at 8–12 weeks of age. Mice were exposed to dilute mainstream cigarette smoke (CS) for 1 hour, twice per day, for 3 days, as described [31]. Resolvin D1 (7S,8R,17S-trihydroxy-4Z,9E,11E,13Z,15E,19Z-docosahexaenoic acid) and 17R-RvD1 (7S,8R,17R-trihydroxy-4Z,9E,11E,13Z,15E,19Z-docosahexaenoic acid) were obtained from Cayman Chemical. In the pre-treatment exposure model, 100 ng RvD1 in 40 µl saline was given by oropharyngeal aspiration on day 0, and 1 hour prior to the first smoke exposure on days 1, 2 and 3. Control animals were treated with saline vehicle. Smoke exposures were quantified by determining total suspended particulate matter (TSP) by gravimetric sampling. The smoke concentration was set at a nominal value of 450 mg/m^3^ TSP by adjusting the number of cigarettes loaded onto the carousel and the flow rate of the dilution air. Control animals were exposed to filtered air in identical equipment on the same schedule. Mice were euthanized on day 4 and cells and tissues were harvested for analysis. To analyze the resolution of active inflammation, mice were exposed to CS for 2 hours per day on days 1–3. On day 3, after the final smoke exposure, and on days 4 and 5, the mice were treated with vehicle or 100 ng RvD1 by oropharyngeal aspiration as described. The mice were euthanized and analyzed on day 4 and 6.

### Analysis of Bronchoalveolar Lavage Fluid (BALF) and Lung Tissues

BALF was collected from smoke-exposed mice and analyzed as previously described [31]. The right lung was frozen in liquid nitrogen for later analysis. Total protein in BALF was determined by the bicinchoninic acid (BCA) colorimetric assay (Thermo Scientific, Rockford, IL). The right lung was homogenized in RIPA buffer as described [32], and lung proteins were analyzed for expression of COX-2 (Clone CX229, Cayman Chemical, Ann Arbor, MI), Arg-1 (H-52; Santa Cruz Biotechnologies, Santa Cruz, CA) and total actin (CP01; Calbiochem, San Diego) by Western blotting. In some experiments, the lungs were inflated and fixed with 10% neutral buffered formalin without undergoing lavage. Tissues were embedded with paraffin, sectioned (5 µm), and stained with hematoxylin and eosin (H&E).

### Immunohistochemistry

Lung-infiltrating neutrophils were stained with rat anti-mouse Ly-6B.2 (MCA771GA, Serotec, Oxford, UK) and HRP-conjugated goat anti-rat antibodies, followed by visualization with AEC chromagen (Vector Lab, Burlingame, CA) and hematoxylin counter staining.

### Analysis of Cytokines and Chemokines

Human IL-6, IL-8 and MCP-1 were measured in cell culture media by commercial ELISA according to the manufacturer’s instructions (R&D Systems). Prostaglandin E_2_ (PGE_2_) was measured by competitive enzyme immunoassay (EIA) using commercially available reagents as described(Cayman Chemical) [33]. Mouse cytokines and chemokines were measured in BALF by ELISA (MIP-2, KC; R&D Systems) or by bead-based multiplex analysis of lung homogenates (IL-1β, IL-6, MCP-1, IFN-γ and IL-10; Millipore, Billerica, MA) using a Luminex FlexMAP3D instrument (Luminex, Austin, TX). Results are expressed as picograms of cytokine per milligram of total protein in the homogenate.

### Assessment of Macrophage Phagocytosis of Neutrophils

Lungs were harvested from mice exposed to smoke and treated with RvD1 as described above. One lobe was digested with collagenase and a single cell suspension was prepared. Total lung macrophages were isolated by CD11b magnetic bead separation (Miltenyi Biotech, Cambridge, MA) [34]. The cells were then labeled with FITC-conjugated anti-mouse F4/80 antibody (BM8; Invitrogen; Camarillo, CA) for 30 minutes, then fixed with 4% of paraformaldehyde and permeabilized with 0.1% Triton X-100. Ingested neutrophils were labeled by staining permeabilized cells with PE-conjugated anti-mouse Gr-1 (RB6-8C5; BD Pharmingen) antibodies for 30 minutes and analyzed by flow cytometry (FACSCanto II; BD Biosciences, San Jose, CA). Forward and side scatter was first used to exclude doublets or macrophages that are attached to but did not fully engulf neutrophils. Cells were then gated based on F4/80 positive staining. The frequency of cells that are F4/80 and GR-1 double positive was then quantified.

### Macrophage Phagocytic Activity *in vitro*

Alveolar macrophages were harvested from naïve mice by lavage in PBS buffer containing 0.6 mM EDTA and glucose (0.1%). Macrophages were activated with 1 ng/ml IL-1β for 30 minutes and then incubated with RvD1 (100 nM in 0.1% ethanol) or 0.1% ethanol alone for an additional 30 minutes before the addition of a 10-fold excess of FITC-conjugated latex microspheres for 1 hour. Uptake of FITC-labeled latex microspheres was measured by flow cytometry (FACSCanto II, BD Biosciences).

### Characterization of Alveolar Macrophages

Lungs were lavaged extensively (0.5 ml for 5–10 times) with PBS containing 0.1% glucose to collect lung macrophages. Collected cell suspensions were enriched for macrophage by adherence on tissue culture-treated plastic dishes for 1 hour in minimum essential medium (MEM; Gibco, Camarillo, CA) containing 10% fetal bovine serum. Non-adherent cells were discarded by gently washing twice with PBS. RNA was extracted from the enriched adherent macrophages by RNeasy kit according to manufacturer’s instructions (Qiagen; Valencia, CA). RNA (1 µg) was reverse-transcribed using the iScript cDNA synthesis Kit (BioRad; Hercules, CA) and the cDNA was subjected to quantitative real-time PCR using SYBR GreenER in a BioRad iQ5 cycler. In brief, a 20 µl mixture was used containing 10 µl iQ SYBR Green Supermix, 0.5 µl forward and reverse primer, 7.5 µl sterile water, and 2 µl of the 1∶5 diluted cDNA template. The real-time PCR was performed under the following conditions: 1 cycle at 95°C for 3 minutes, then 40 cycles at 95°C for 10 seconds, 55°C for 30 seconds, 95°C for 1 minute followed by 55°C for 1 min. The intron-spanning primers were designed by using sequence information from the NCBI database. The Ct values were normalized to the endogenous control (18sRNA). Primer sequences are listed below:

m18sRNA: 5′-GCTTGCTCGCGCTTCCTTACCT-3′

m18sRNA: 5′ TCACTGTACCGGCCGTGCGTA-3′

mArg1-F: 5′- CAGAAGAATGGAAGAGTCAG–3′

mArg1-R: 5′- CAGATATGCAGGCAGGGAGTCACC -3′

mMrc1-F: 5′- CTCTGTTCAGCTATTGGACGC -3′

mMrc1-R: 5′- CGGAATTTCTGGGATTCAGCTTC-3′

miNOS-F: 5′- GTTCTCAGCCCAACAATACAAGA -3′

miNOS-R: 5′- GTGGACGGGTCGATGTCAC -3′

mTNFα-F: 5′- GACGTGGAACTGGCAGAAGAG -3′

mTNFα -R: 5′- TTGGTGGTTTGTGAGTGTGAG -3′

mIL-10-F: 5′- GCTCTTACTGACTGGCATGAG -3′

mIL-10-R:5′- CGCAGCTCTAGGAGCATGTG -3′

### Statistical Analysis

Results are reported as the mean ± SEM or mean ± SD as indicated. T-test (two tailed) or one-way and two-way analysis of variance (ANOVA) with Bonferroni multiple comparisons were performed using GraphPad Prism version 5.0d for Mac (GraphPad Software, La Jolla California USA, www.graphpad.com). A P value <0.05 was considered significant.

## Results

### RvD1 Inhibits IL-1β-induced and CSE-induced Cytokine and Chemokine Production in Primary Human Lung Fibroblasts

**Figure 1:**
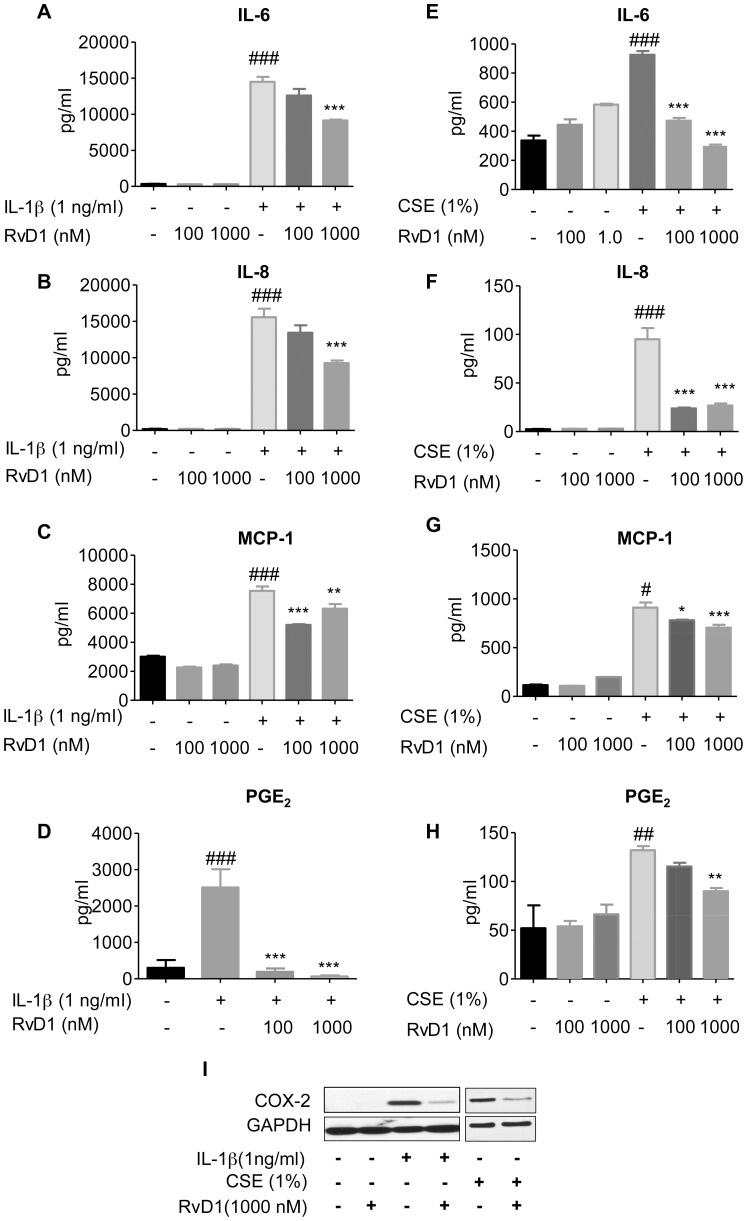
RvD1 inhibits IL-1β-induced and CSE-induced cytokine and chemokine production from primary human lung fibroblasts. Primary human lung fibroblasts were pre-treated with the indicated concentrations of RvD1 for 24 hours then stimulated with IL-1β (1 ng/ml) or cigarette smoke extract (CSE, 1%) for an additional 24 hours. Culture media from IL-1β-treated cells (**A–D**) or CSE-treated cells (**E–H**) were subjected to ELISA and EIA to detect various pro-inflammatory mediators as described in the *Methods*. Data shown are mean ± SD for n = 4 replicate cultures, from one representative experiment of 3 performed. COX-2 and GAPDH expression (**I**) were determined in cell lysates by Western blotting. Blots shown are representative of three independent experiments, and the lanes were run non-contiguously on the same gel. ***P<0.001, **p<0.01 *P<0.05 compared to IL-1β-treated or CSE-treated cultures without RvD1, ^###^P<0.001 versus vehicle-treated control cultures by one-way ANOVA with Bonferroni post-tests.

Lung fibroblasts produce many pro-inflammatory molecules and amplify inflammatory cascades following exposure to pro-inflammatory insults including cigarette smoke and IL-1β [35]. To evaluate the ability of RvD1 to suppress inflammatory signaling in lung fibroblasts, primary human lung fibroblasts were pre-treated for 24 hours with the indicated concentrations of RvD1 then stimulated with cigarette smoke extract (CSE) or IL-1β for an additional 24 hours. IL-1β strongly induced production of pro-inflammatory mediators including IL-6, IL-8, MCP-1 and PGE_2_ (Figure 1). RvD1 significantly inhibited the production of MCP-1 and PGE_2_ at 100 nM, and production of IL-6, IL-8, MCP-1 and PGE_2_ at 1000 nM. CSE also induced these pro-inflammatory mediators in human lung fibroblasts, although at lower levels than IL-1β. IL-6, IL-8, MCP-1 and PGE_2_ were significantly attenuated by RvD1 treatment (Figure 1, panels E, F, G and H). The pro-inflammatory enzyme COX-2 is responsible for PGE_2_ production and is elevated in a variety of inflammatory pulmonary diseases include COPD [36]. IL-1β and CSE both induced COX-2 expression in human lung fibroblasts. Exposure to RvD1 (1000 nM) significantly attenuated COX-2 expression (Figure 1I), suggesting a potent anti-inflammatory function for RvD1 in human lung fibroblasts.

### RvD1 Inhibits Production of Inflammatory Mediators in Monocytes and Lung Epithelial Cells

**Figure 2:**
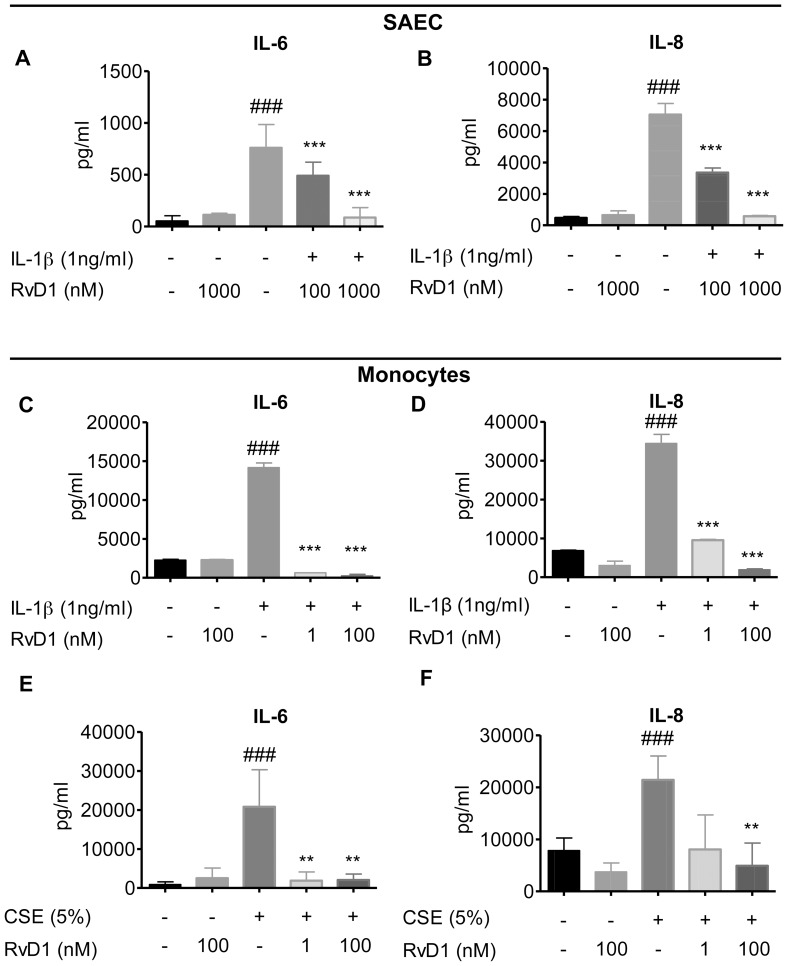
RvD1 inhibits IL-1β and CSE-induced cytokine production from primary human small airway epithelial cells and monocytes. Primary SAEC (**A–B**) were pre-treated with indicated concentrations of RvD1 for 24 hours then stimulated with IL-1β (1 ng/ml) for an additional 24 hours. Culture media was collected and levels of IL-6 (**A**) and IL-8 (**B**) were determined by ELISA. Data shown are mean ± SD of triplicate cultures, from one representative experiment of 3 performed. Blood-derived monocytes (**C–F**) were pre-treated with indicated concentrations of RvD1 for 24 hours then stimulated with IL-1β (1 ng/ml) or CSE (5%) for an additional 24 hours. Culture media was collected and levels of IL-6 (**C, E**) and IL-8 (**D, F**) were determined by ELISA. Data shown are mean ± SEM for three individual donors. ^##^p<0.01, ^###^p<0.001 compared to vehicle-treated control cultures. **p<0.01, ***p<0.001 compared to IL-1β or CSE without RvD1, using one-way ANOVA with Bonferroni post-tests.

Macrophages and epithelial cells are important sources of inflammatory cytokines in cigarette smoke-exposed lungs. To evaluate the effect of RvD1 on these cell types, human primary small airway epithelial cells (SAECs) and blood-derived monocytes were pre-treated with the indicated concentrations of RvD1 before the addition of IL-1β or CSE for an additional 24 hours. RvD1 significantly dampened production of IL-6 and IL-8 from SAEC treated with IL-1β (Figure 2A–B). In our hands, IL-1β did not stimulate release of PGE_2_, and CSE did not stimulate production of IL-6 or IL-8 from SAEC (data not shown). Both IL-1β and CSE induced expression of IL-6 and IL-8 from human monocytes. A one-hour pre-treatment with RvD1 significantly inhibited production of IL-6 and IL-8 in a dose-dependent manner (Figure 2C–F). In these experiments the monocytes produced high background levels of PGE_2_ which was not significantly increased further by CSE, therefore an effect of RvD1 on PGE_2_ production could not be determined (data not shown). Interestingly, monocytes were more resistant to CSE than fibroblasts and required treatment with 5% CSE to induce a strong response, but were more sensitive to RvD1, with potent inhibition seen at 10 nM (Figure 2C–F).

### RvD1 Attenuates Cigarette Smoke-induced Inflammation *in vivo*

**Figure 3:**
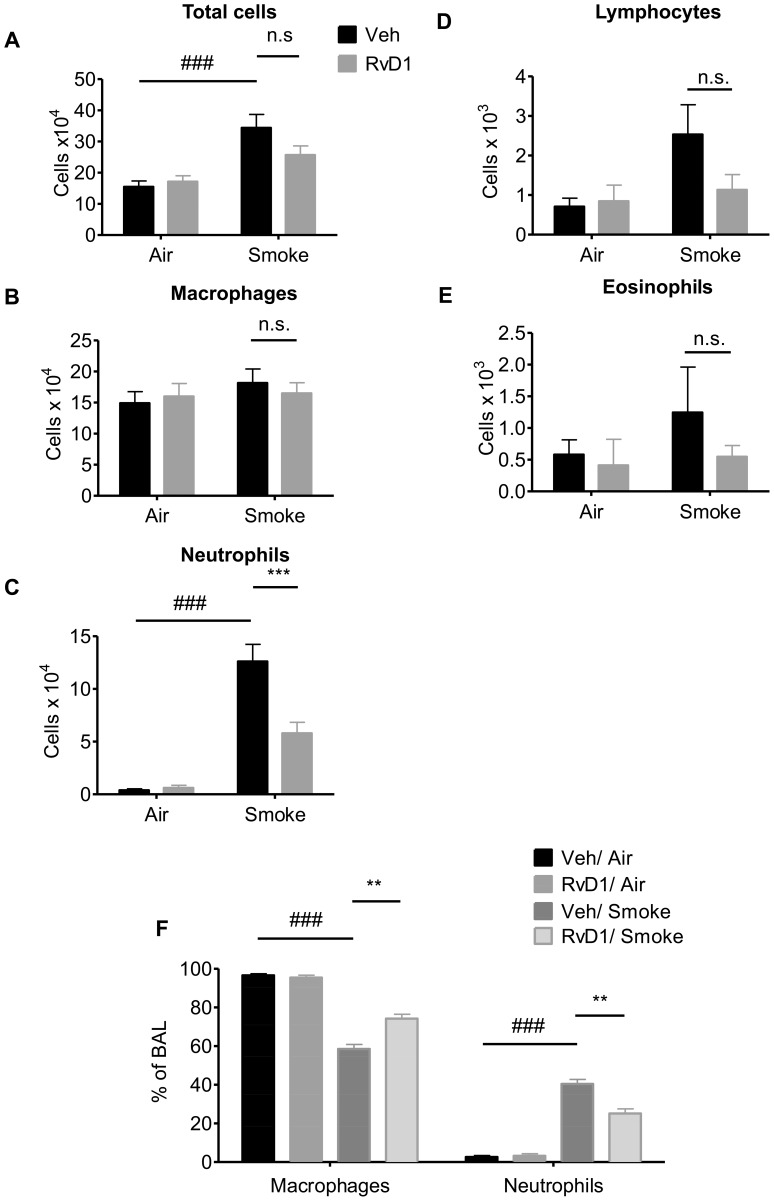
RvD1 attenuates CS-induced inflammation *in vivo.* Mice were treated with 100 ng RvD1 (grey bars) or saline vehicle (Veh, black bars) by inhalation 1 hour prior to cigarette smoke, daily for 3 days. Mice were euthanized at day 4, and differential cell counts were performed on BALF cells. Total cell number (**A**), number of macrophages (**B**), neutrophils (**C**), lymphocytes (**D**), eosinophils (**E**) and percentage of macrophages and neutrophils (**F**) are reported. Data are shown as mean ± SEM for n  = 6–7 mice per group from one of 3 independent experiments. ***P<0.001, versus smoke-exposed mice by two-way ANOVA with Bonferroni post-tests. ^###^P<0.001, versus air-exposed control mice by one-way ANOVA with Bonferroni post-tests.

Based on our *in vitro* results, we hypothesized that administration of RvD1 would attenuate cigarette smoke-induced acute lung inflammation *in vivo*. Mice received 100 ng RvD1 by inhalation 1 hour prior to cigarette smoke exposure daily for 3 days. On day 4, the mice were euthanized, bronchoalveolar lavage fluid (BALF) was collected, and inflammation was evaluated by differential cell counting. Cigarette smoke (CS) exposure induced significant increases in total BAL cells, neutrophils and lymphocytes, but not macrophages or eosinophils (Figure 3), consistent with prior experience with this model [37], [38]. The increase in total cell number is almost entirely due to recruitment of neutrophils (Figure 3C). CS-exposed mice pre-treated with RvD1 had significantly fewer neutrophils in BALF compared to vehicle-treated controls, expressed either as neutrophil number (Figure 3C) or percentage (Figure 3F) of total cells. There was no change in the absolute numbers of macrophages between CS and CS+RvD1-treated mice (Figure 3B). As noted, RvD1 had no significant impact on the numbers of BAL lymphocytes (Figure 3D) and eosinophils (Figure 3E). Interestingly, while total macrophage numbers were not changed, there was a significant increase in the percentage of macrophages in the BALF with RvD1 treatment (Figure 3F).

### RvD1 Reduces Cigarette Smoke-induced Neutrophilic Lung Inflammation

**Figure 4:**
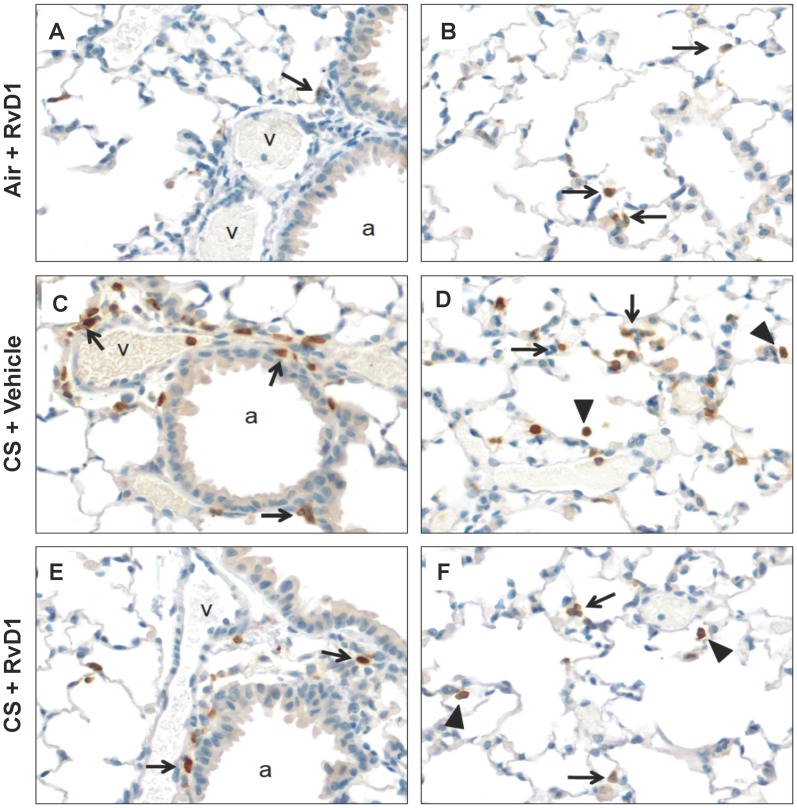
RvD1 reduces CS-induced neutrophilic lung inflammation. Lungs from mice that had *not* been lavaged were inflated and fixed with neutral buffered formalin, and 5 µm paraffin sections were stained with a neutrophil-specific antibody (brown) and counter-stained with hematoxylin. An occasional neutrophil was observed in air-exposed mice treated with RvD1 (**A, B**), similar to air-exposed mice treated with vehicle (not shown). CS induces a neutrophilic inflammation around vessels and airways (**C**) and in the parenchyma (**D**), with neutrophils prominent in the interstitial spaces (arrows) and the alveoli (solid triangles). RvD1 treatment reduces perivascular and peribronchial (**E**) as well as parenchymal (**F**) accumulation of neutrophils. A, airway; v, vessel. Arrows, interstitial neutrophils; solid triangles, neutrophils in airspaces.

The location of infiltrating neutrophils in lung tissues was determined by immunohistochemical staining for the neutrophil specific marker Ly-6B.2. In RvD1-treated, air-expose lung, neutrophils were sparsely detected (Figure 4A and 4B). Acute smoke exposure causes accumulation of neutrophils around vessels (v), airways (a) (Figure 4C, arrows) and in the parenchyma (Figure 4D), with neutrophils prominent in the interstitial spaces (Figure 4D, arrows) and the alveoli (Figure 4D, solid triangles). RvD1 reduces the appearance of CS-induced neutrophils in lung tissue consistent with the results of differential cell counts in BALF that RvD1 reduces neutrophil accumulation in the lung.

### RvD1 Decreases Production of Pro-inflammatory Mediators

**Figure 5:**
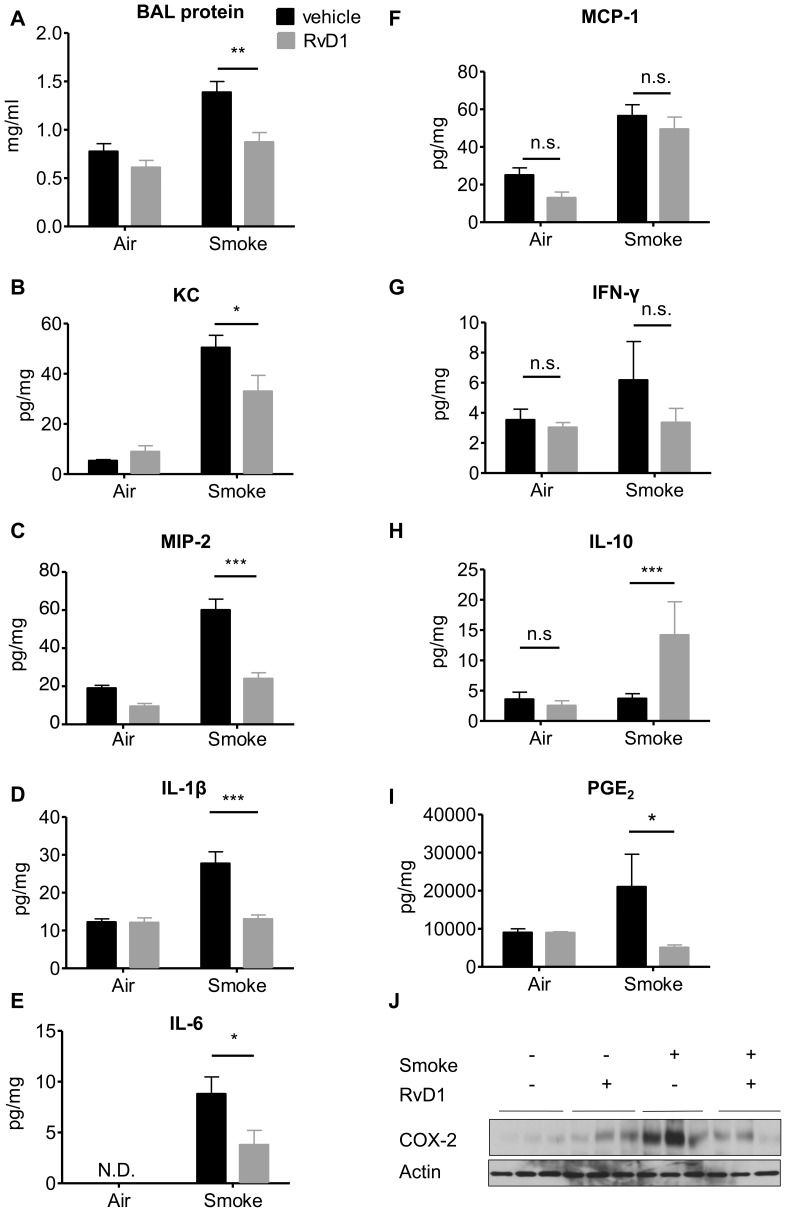
RvD1 decreases production of pro-inflammatory mediators in BALF and total lung homogenate but increases IL-10. Total protein (**A**) in BALF was measured by BCA assay. Neutrophil chemoattractants KC (**B**) and MIP-2 (**C**), IL-1β (**D**), IL-6 (**E**), MCP-1 (**F**), IFN-γ (**G**), PGE_2_ (**H**) and IL-10 (**I**) were determined in lung homogenates by multiplex assay or EIA and normalized to total protein concentration. N.D., not detected. Data are shown as mean ± SEM for n = 6–8 mice per group and are representative of 3 independent experiments. *P<0.05, **P<0.01, ***P<0.001 versus smoke-exposed mice by two-way ANOVA with Bonferroni post-tests. ^###^P<0.001 compared to air-exposed control mice by one-way ANOVA with Bonferroni post-tests. Expression of COX-2 and total actin (**J**) were determined in whole lung homogenates by Western blotting using COX-2 and total actin specific antibodies. Each lane represents an individual mouse.

Levels of pro-inflammatory cytokine and chemokine were determined in BALF and lung homogenates of smoke-exposed mice. Total protein in BALF, an indication of vascular leakage and edema, was also increased with CS exposure and significantly reduced with RvD1 treatment (Figure 5A). Acute cigarette smoke exposure induces the expression of neutrophil chemoattractants KC and MIP-2, which was significantly inhibited by RvD1 (Figure 5, panels B and C). CS exposure also resulted in increased production of pro-inflammatory cytokines including IL-1β, IL-6, and MCP-1. RvD1 markedly decreased IL-1β and IL-6 but not MCP-1 (Figure 5, panels D-F). IFN-γ content was increased with CS and decreased with CS+RvD1 treatment, however the difference was not significant (Figure 5G). Interestingly, the anti-inflammatory cytokine IL-10 was significantly increased in lungs from CS-exposed mice pre-treated with RvD1 (Figure 5H). We also evaluated expression of COX-2 protein and PGE_2_ levels in lung homogenates, which showed results consistent with our *in vitro* findings that CS increases PGE_2_ production and COX-2 expression, which is strongly inhibited by RvD1 (Figure 5I and 5J, respectively).

### 17R-RvD1 Promotes the Resolution of Acute Inflammation Induced by CS Exposure

**Figure 6:**
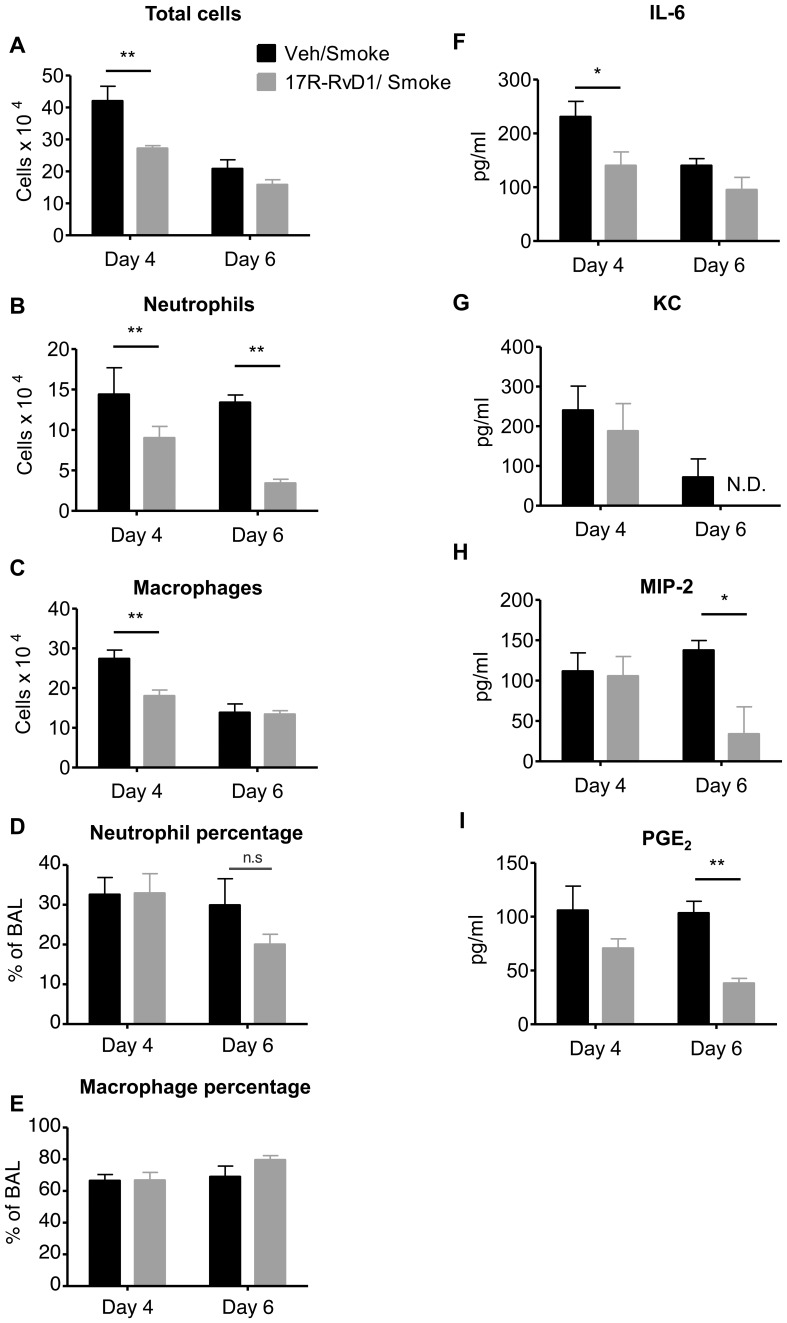
17R-RvD1 promotes the resolution of acute lung inflammation induced by CS exposure. Mice were exposed to CS exposure on days 1, 2 and 3. The mice received 100 ng 17R-RvD1 (grey bars) or saline vehicle (Veh, black bars) by inhalation on day 3 (1 hour after the last cigarette smoke exposure), day 4 and day 5. Mice were euthanized at days 4 and 6 and differential cell counts were performed on BAL cells. Total cell number (**A**), number of neutrophils (**B**), and macrophages (**C**), percentage of neutrophils (D) and macrophages (E) are reported. BALF was collected and analyzed by ELISA or EIA for IL-6 (**F**), KC (**G**), MIP-2 (**H**) and PGE_2_ (**I**). Data are shown as mean ± SEM for n  = 4–5 mice per group. N.D., not detected. *P<0.05, **P<0.01 compared to Vehicle/Smoke mice by two-way ANOVA with Bonferroni post-tests.

Resolvins are reported to be pro-resolving as well as anti-inflammatory [39]. To evaluate whether resolvins could accelerate the resolution of CS-induced inflammation, we exposed mice to CS for 3 days, and initiated resolvin treatment after the final smoke exposure. For these experiments, we used the 17R epimer of RvD1 (17R-RvD1) as it is less susceptible to *in vivo* inactivation [4]. RvD1 and 17R-RvD1 bind the same receptor and have similar activities *in vitro* [21]. Mice were euthanized on day 4 (after a single 17R-RvD1 treatment on day 3) or day 6 (after receiving 17R-RvD1 on days 3, 4 and 5). A single dose of 17R-RvD1 accelerated the resolution of acute CS-induced inflammation, with significant reductions in total BAL cells, macrophages and neutrophils on day 4 (Figure 6, panels A, B and C). Although the number of inflammatory cells was significantly reduced by 17R-RvD1, the percentages of neutrophils and macrophages did not significantly change, as the number of macrophages, neutrophils, and total cell numbers were reduced together (Figure 6E, F). Inflammation on day 6 was also reduced with 17R-RvD1, although less dramatically than on day 4. 17R-RvD1 also significantly reduced production of the pro-inflammatory cytokine IL-6 (Figure 6F), neutrophil chemokines KC and MIP-2 (Figure 6G and H, respectively) and PGE_2_ (Figure 6I).

### RvD1 Enhances the Clearance of Neutrophils *in vivo* and *in vitro*

**Figure 7:**
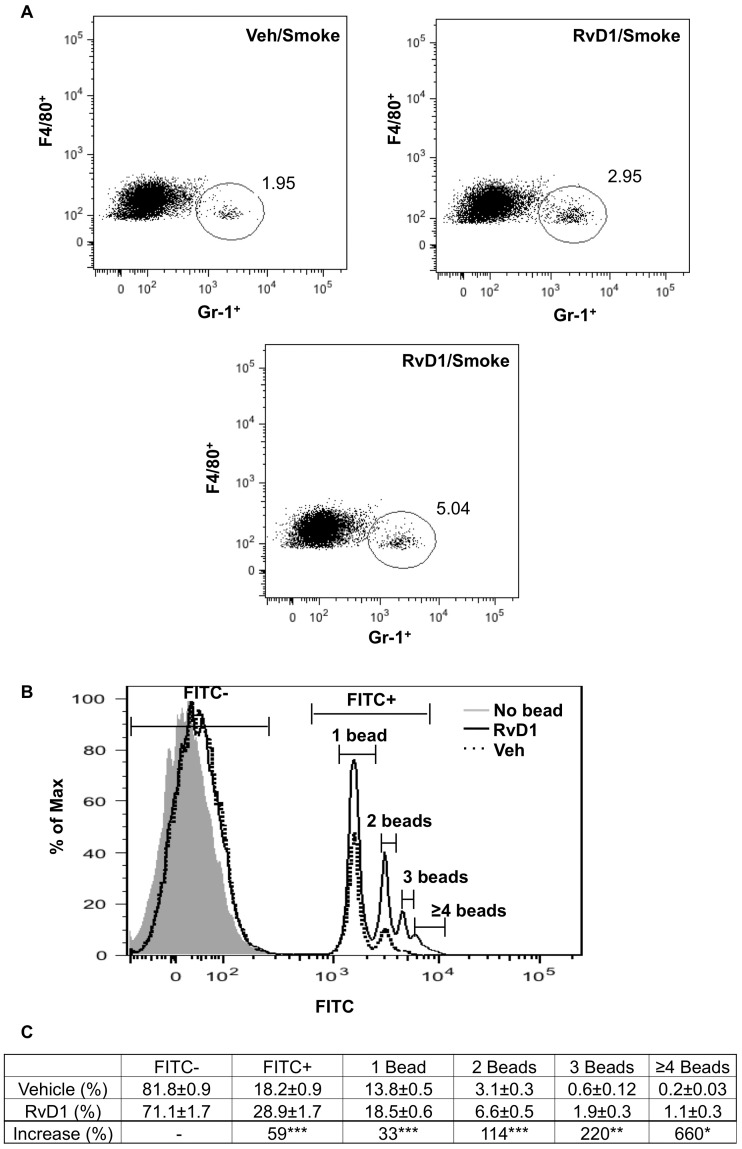
RvD1 enhances macrophage phagocytosis *in vivo* and *ex vivo.* Mice were exposed to air or CS with or without RvD1 as described. Lungs were digested and macrophages were isolated as described in Methods. The percentage of F4/80/Gr-1 double positive cells was quantified by flow cytometry. (**A**) Dot plots shown are from one vehicle control mouse and two RvD1-treated mice. (**B**) BAL cells were harvested from naïve mice and incubated *ex vivo* with 1 ng/ml IL-1β and 100 nM RvD1 for 30 minutes prior to the addition of FITC-labeled latex beads. Percentage of F4/80^+^ macrophages that ingested FITC-latex beads was quantified by flow cytometry. Representative histogram of one out of 6 mice is shown. (**C**) Mean ± SEM is shown for 6 animals from two independent experiments. *p<0.05, **p<0.01, ***p<0.001, Student’s t-test.

We hypothesized that one mechanism by which RvD1 promotes resolution of CS-induced inflammation is by increasing the phagocytosis of neutrophils by lung macrophages. To investigate this mechanism, lung macrophages were harvested from CS-exposed mice, fixed and permeabilized, then co-stained for the macrophage marker, F4/80, and the neutrophil marker Gr-1. Treatment with RvD1 increased the percentage of F4/80 and Gr-1 double positive cells, suggesting that clearance of apoptotic neutrophils is enhanced in the presence of RvD1 (Figure 7A). We also investigated whether RvD1 increased macrophage phagocytosis *ex vivo*. Alveolar macrophages were isolated from naïve mice and stimulated with IL-1β (1 ng/ml) for 30 minutes, followed by RvD1 or vehicle for and additional 1 hour. The phagocytic ability of the macrophages was assessed using FITC-labeled latex beads and analyzed by flow cytometry. RvD1 enhanced overall bead uptake by 59% (Figure 7, panel B and C). In addition to increasing the percentage of macrophages that had phagocytosed at least one bead, RvD1 also increased the number of beads phagocytosed per cell, with a 220% increase in the percentage of cells ingesting 3 and a 660% increase in the percentage of cells ingesting 4 or more beads (Figure 7C).

### RvD1 Drives Polarization of Alternatively Activated Macrophages

**Figure 8:**
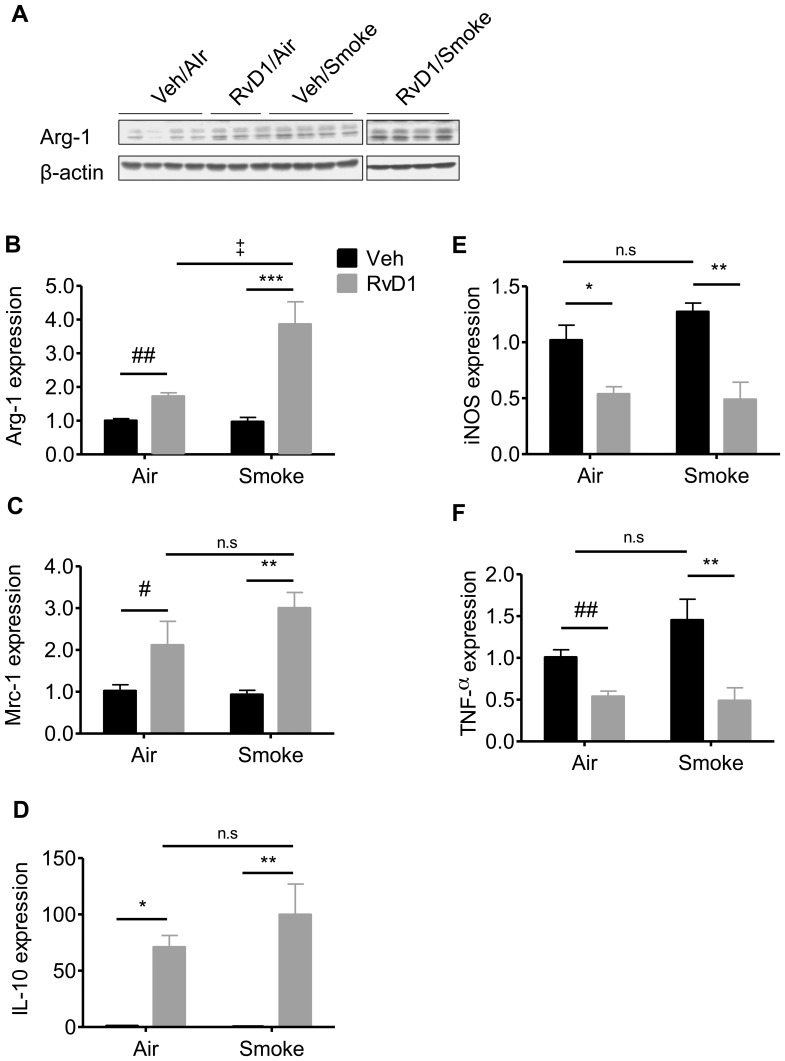
RvD1 drives polarization of alternatively activated macrophages. Mice were exposed to air or CS with or without RvD1 as described. (**A**) Expression of Arg-1 was measured in whole lung homogenates by Western blotting. Each lane represents one animal. **B–F:** Alveolar macrophages were enriched from BAL cells by adherence and total RNA was extracted and subjected to quantitative RT-PCR to measure mRNA levels of *Arg1* (**B**), *Mrc1* (**C**), *IL-10* (**D**), *iNOS* (**E**) and *TNFα* (**F**). Mean ± SEM is shown, normalized to 18S rRNA. Each group n = 6 for Arg1, Mrc1 and IL-10; n = 3 for iNOS and TNFα. ^#^P<0.05, ^##^P<0.01, compared to Vehicle/Air animal by one-way ANOVA with Bonferroni post-tests; *P<0.05, **P<0.01, ***P<0.001 versus Vehicle/Smoke animal by two-way ANOVA with Bonferroni post-test. ‡P<0.05 between RvD1/Air and RvD1/Smoke, ANOVA.

Given the increased IL-10 production and increased phagocytic activity seen with RvD1 treatment, we hypothesized that RvD1 drives macrophage polarization to an M2 phenotype. RvD1 increased protein levels of arginase I (*Arg1*), a marker of M2 macrophages, in total lung homogenates (Figure 8A). We also extracted RNA from lung macrophages from mice exposed to air or CS, with or without RvD1 treatment, and determined mRNA levels by quantitative RT-PCR. In macrophages from air-exposed mice, RvD1 induced the M2 genes *Arg1*, mannose receptor 1 (*Mrc1)* and *IL-10* demonstrating that the number of M2 macrophages increased after RvD1 treatment in the absence of inflammatory stimuli (Figure 8B–D). In CS-exposed mice, RvD1 significantly increased mRNA expression of *Arg1*, *Mrc1* and *IL-10* from alveolar macrophages compared to vehicle-treated CS-exposed mice (Figure 8, panels B-D). RvD1 significantly reduced mRNA expression of *iNOS* and *TNF-α* (Figure 8, panels E and F), two mediators highly expressed in classically-activated (M1) macrophages. Taken together, these results clearly demonstrate that RvD1 significantly augments the polarization of alveolar macrophages to the M2 phenotype.

## Discussion

Human lungs are exposed daily to thousands of liters of air containing numerous environmental insults and pro-inflammatory stimuli including dust, pollen, mold, ozone and hydrocarbon pollutants. Given this, regulating the balance between pro-inflammatory and pro-resolving signaling pathways is crucial to maintain normal lung homeostasis [39]. Cigarette smoke is a powerful pro-inflammatory stimulus and a leading cause of lung, cardiovascular and other diseases [1], [15]. Resolvin D1 is a member of a novel class of lipid mediators with anti-inflammatory and pro-resolving functions [3]. Here, we report that RvD1 is a potent inhibitor of cigarette smoke-induced pro-inflammatory signaling in human lung cells *in vitro* and has potent anti-inflammatory and pro-resolving properties in a mouse model of acute cigarette smoke-induced lung inflammation.

We first investigated the response of primary cells to RvD1 *in vitro*. RvD1 significantly reduced inflammatory responses in primary human lung fibroblasts and blood-derived monocytes using both IL-1β and CSE as inflammatory stimuli, and from human small airway epithelial cells stimulated with IL-1β (Figures 1 and 2). We used IL-1β as a model stimulus because we and others have shown that CS exposure upregulates IL-1β levels in both mice and humans exposed to cigarette smoke (Figure 4) [40]. Therefore, lung cells are likely exposed to IL-1β following smoke exposure *in vivo*. Cigarette smoke extract is widely used as an *in vitro* analog to inhalation of smoke but an exact equivalence between *in vitro* and *in vivo* exposures can not be determined [41]. It is interesting to note that RvD1 abolished IL-1β-induced PGE_2_ production in lung fibroblasts and inhibited PGE_2_ production *in vivo*. The role of PGE_2_ in regulating inflammation is complex, as PGE_2_ has both pro-inflammatory and pro-resolving properties. PGE_2_ is a potent neutrophil chemokine, and is upregulated by cigarette smoke (Figure 5) [12], [42], [43]. However, PGE_2_ also has anti-inflammatory properties in neutrophils, and is involved in activating lipid mediator class switching between pro-inflammatory and pro-resolving mediators [44], [45]. Here, RvD1 has a net anti-inflammatory effect, perhaps because its pro-inflammatory effects are suppressed by RvD1, while its pro-resolving effects are replaced by exogenous RvD1.

Cigarette smoking causes both acute and chronic lung inflammation with prominent involvement of neutrophils. Neutrophilic airway inflammation is one of the key underlying pathophysiological processes in the development of COPD [46]. Recently, it was shown that cigarette smoke disrupts the normal resolution of neutrophilic lung inflammation by reducing neutrophil apoptosis and efferocytosis [47]. Impairment of neutrophil clearance can lead to irreversible local tissue damage via production of mediators including neutrophil elastase and reactive oxygen species [48]. Here we report that RvD1 dampens neutrophil recruitment and enhances neutrophil clearance in acute CS-induced inflammation. These results suggest that RvD1 or 17R-RvD1 might be useful as therapies to reduce chronic inflammation and lung injury in diseases of chronic smoke exposure such as COPD.

Alternatively activated or type II macrophages (M2) are immunoregulatory cells that possess anti-inflammatory properties and eliminate tissue debris and apoptotic bodies via phagocytosis. Previous reports have shown that RvE1 and RvD2 enhanced macrophage phagocytosis of bacterial ligands and apoptotic neutrophils in sepsis and asthma models [49], [50]. Here we show that RvD1 promotes differentiation of pro-resolving macrophages that have enhanced phagocytic activity *in vitro* and *in vivo* (Figure 7 and 8). It was recently reported that RvD1 regulates pro- and anti-inflammatory micro RNAs via signaling through its G-protein receptors ALX and GPR32, resulting in increased production of IL-10 and decreased NF-κB activity [51]. Additionally, it is known that IL-10 promotes the M2 phenotype [14], [16]. These results, combined with our findings that IL-10 is elevated in RvD1-treated lungs (Figure 5H) and alveolar macrophages (Figure 8D), demonstrate that RvD1 and IL-10 enhance M2 polarization by a positive feedback mechanism initiated by RvD1-mediated G-protein signaling.

Although neutrophil chemokines (MIP-2, KC) and broad-spectrum pro-inflammatory signals (IL-1β, IL-6) were strongly inhibited by RvD1, the macrophage chemoattractant MCP-1, which was also induced by CS, was not reduced by RvD1 in mouse lung. Interestingly, it has been reported that MCP-1 enhances efferocytosis by alveolar macrophages, and that MCP-1 is important for the resolution of inflammation [52]. This is consistent with our observations that macrophage numbers were not reduced by RvD1 and that RvD1 enhances efferocytosis (Figure 7). RvD1 also up-regulated production of IL-10, a potent immunoregulatory cytokine that is reported to be essential for the resolution of lung inflammation [53] and which is significantly decreased in sputum samples from COPD patients [54], [55]. Taken together, this suggests that RvD1 is not an indiscriminate anti-inflammatory agent, but rather selectively activates specific anti-inflammatory and pro-resolving pathways that include inhibition of neutrophilic inflammation and the activation of a subset of anti-inflammatory, pro-resolving macrophages.

Smoking-related inflammation of the lungs, cardiovascular system and other organs persists for months or even years after smoking cessation, and COPD not only persists but also may continue to worsen [1], [2]. This suggests that development of novel therapeutics should be focused on promoting resolution, rather than preventing inflammation. Here, a single dose of 17R-RvD1 given after the final smoke exposure resulted in accelerated reduction in BAL neutrophils and IL-6 (Figure 6). The pro-resolving activity was maintained for at least 3 days, with continued reductions in neutrophils and key pro-inflammatory mediators. These results are similar to results recently reported from Dr. Serhan’s laboratory that RvD1 and 17R-RvD1 accelerated the resolution of allergic airway inflammation in the mouse ovalbumin model [25]. 17R-RvD1 is an alternate epimer of RvD1 produced endogenously in the presence of aspirin, which acetylates the COX-2 resulting in a shift from the 17S to the 17R epimer [21]. The 17S epimer is subject to rapid inactivation by the endogenous enzyme 15-prostaglandin dehydrogenase/eicosanoid oxidoreductase (15-PGDH) whereas the 17R epimer is not. While RvD1 was effective at inhibiting inflammation when given immediately prior to smoke exposure, we were concerned that it may not have a long enough half-life to be effective at promoting resolution when given after smoke exposure. 17R-RvD1 has similar biological activity and uses the same receptors [21] but is resistant to 15-PGDH and has a longer *in vivo* half-life [4], [25]. It is also possible that 17R-RvD1 would have been more efficacious than RvD1 in the pre-treatment model (Figures 3, 4, 5), although this has not been tested.

Cigarette smoke is a direct cause of several devastating diseases including COPD for which there are few effective therapeutic agents. Long-term use of glucocorticoids carries risks of infection and other adverse effects potentially outweighing their benefits [56], and many long-term smokers and emphysema patients exhibit steroid resistance [57]. By blocking utilization of arachidonic acid, COX-2 inhibitors not only block pro-inflammatory signals, but also inhibit resolvin biosynthesis [58]. Resolvins offer significant clinical potential because they promote resolution and repair without being immunosuppressive. Here, we have demonstrated that RvD1 possesses potent anti-inflammatory and pro-resolving activity *in vivo* and *in vitro*. Inhalation therapy with RvD1 may offer an alternative approach for treating human inflammatory lung diseases including chronic lung injury and COPD resulting from cigarette smoking and other chronic injuries.
